# A Randomized Field Experiment Using Self-Reflection on School Behavior to Help Students in Secondary School Reach Their Performance Potential

**DOI:** 10.3389/fpsyg.2020.01356

**Published:** 2020-06-30

**Authors:** Eva Feron, Trudie Schils

**Affiliations:** ^1^Organisation of Economic Co-operation and Development (OECD), Centre for Educational Research and Innovation, Paris, France; ^2^Department of Macro, International and Labour Economics, School of Business and Economics, Maastricht University, Maastricht, Netherlands

**Keywords:** underperformance, social–emotional skills, randomized field experiment, school engagement, school performance, secondary education

## Abstract

Recent policy reports documented that a growing group of students in secondary education could perform better given their expected performance. Studies showed that school performance is related to a range of social–emotional factors, including self-awareness, self-management, social awareness, and responsible decision making. However, experimental studies in schools on the relation between these factors and school performance are scarce, and results are mixed. This study used a randomized field experiment to examine whether self-reflection on school behavior of underperforming secondary school students affected their school performance [grade point average (GPA)], school engagement, and self-concept. The sample comprised 337 ninth-grade students (*M* = 15.74 years old; *SD* = 0.58) from 18 secondary schools in Netherlands. The intervention was designed in co-creation with teachers, to be as close to school practice as possible. Underperformance was measured using achievement test scores from both primary and secondary school, supplemented with teacher and parental assessments. Different model specifications were estimated to perform the analyses and test for robustness of findings. The results showed that, for treatment compliance, students with higher school motivation were approximately 29% more likely to comply. Students who reported higher levels of self-concept of school tasks were 17% less likely to comply. No significant effects of the treatment were observed on students’ GPA, school motivation, hours spent on homework, or self-concept of school tasks. The treatment showed a negative effect on self-concept of leadership skills.

## Introduction

### The Importance of Social–Emotional Skills for School Performance

School performance is about more than just cognitive ability in the domains of, for example, math and reading. It includes the development of social–emotional skills, or the ability to regulate one’s thoughts, emotions, and behavior. This includes empathy, self-efficacy, motivation, self-concept, collaboration, and leadership skills (OECD, 2018). Several psychological theories addressed the relation between such skills and school performance. For example, social cognition models, among which expectancy-value models and achievement-motivation models, argue that students’ achievement motivation and school performance are affected by their goal-setting behavior, as well as their expectations and perceptions or beliefs about their competences and about the difficulty of the tasks they are confronted with (see Wigfield and Cambria, 2010 for an elaborate overview of such models). When students have positive beliefs about their own capabilities in relation to the task they are confronted with and are able to set realistic achievement goals, they are more likely to be motivated to start with the task and to persist when they encounter any difficulties. Consequently, they are expected to perform better at the task, compared to students who have negative beliefs about their own capabilities, or those that set unrealistic goals. This also relates to theories about self-regulatory mechanisms that address aspects of school performance related to students’ task preparation, including goal setting and schematic organization, or students’ performance monitoring and evaluation behavior (cf. Karoly, 1993). Setting realistic goals and reflective monitoring of progress is likely to have positive effects on students’ performance.

Theories of emotional intelligence state the importance of perceiving emotions, using emotions to facilitate thought, and understanding and managing emotions, when explaining variance in students’ performance (cf. Mayer et al., 2004; Talvio and Lonka, 2013). The cognitive activation theory of stress, developed by Ursin and Eriksen (2004), argues that individuals deal differently with stimulations (e.g., an examination in school). They can respond in an active problem-solving manner, or they can respond in a passive way, resulting in avoidance and procrastination. Such coping strategies are likely related to students’ learning behavior in school, their school performance, and school engagement. Using a metatheoretical perspective, Ziegler and Heller (2000) argue that indeed not only factors such as coping with stress, test anxiety, and expectations, but also achievement motivation and learning and work strategies, are among the social–emotional factors that affect the process of school performance.

A number of empirical studies showed that students’ school performance and behavior in later life not only were related to their abilities and knowledge, but was also driven by personality and social–emotional skills (Heckman, 2000; Heckman and Rubinstein, 2001; Carneiro and Heckman, 2003; Heckman et al., 2006, 2014; Heckman and Masterov, 2007; Poropat, 2009, 2014; Cunha et al., 2010; Kautz et al., 2014; Spengler et al., 2015, 2018; Zajacova and Montez, 2017). For example, Cunha et al. (2010) showed that 34% of variation in educational attainment was explained by ability and social–emotional factors (e.g., temperament, social development, behavioral problems, and self-competence), with 16% accounted for by ability and 12% by the social–emotional factors. In addition, Kautz et al. (2014) showed that social–emotional factors predict school performance above and beyond ability.

Empirical studies in the field of educational sciences and psychology yield more information on the exact aspects within social–emotional skills that relate to higher school performance. Several studies showed that aspects such as being able to plan and organize tasks, self-discipline, future goal orientation, self-confidence, daily learning routines, and being able to focus on important tasks were positively related to school performance (Deci and Ryan, 2000; Andriessen et al., 2006; Dietz et al., 2007; Dignath and Büttner, 2008; Lee et al., 2010; Hodis et al., 2011; McClure et al., 2011; Corker and Donnellan, 2012; Spengler et al., 2015). Some studies also explicitly showed that such factors predicted school performance above and beyond cognitive factors (Steinmayr and Spinath, 2009; Kriegbaum et al., 2015; Steinmayr et al., 2019). For example, Steinmayr et al. (2019) showed that, after controlling for students’ intelligence and grades, students’ self-concept of their ability accounted for at least 10% of the variance in academic achievement.

Although many of the empirical studies were of a correlational nature, a limited number of (quasi-)experimental approaches revealed evidence that there is a positive causal relation between social–emotional factors (such as motivation, self-confidence, aspirations, goal orientation, academic self-concept) and student performance (Heckman and Rubinstein, 2001; Eisen et al., 2003; Spinath and Stiensmeier-Pelster, 2003; Machin et al., 2004; Fryer, 2013; Paunesku et al., 2015). Spinath and Stiensmeier-Pelster (2003), for example, showed that having a realistic, rather than a high, academic self-concept mattered for performance. Especially for students with low levels of academic self-concept, learning could be enhanced by focusing and reflecting on individual learning progress and task enjoyment, rather than setting (competitive) performance goals in terms of results.

### Social–Emotional Factors and Underperformance in School

A growing group of students in secondary education could perform better given their learning potential; that is, they show signs of underperformance. This could be related to a multitude of, often interrelated, factors at different levels, such as the student level, teacher level, school level, or factors stemming from the outside-school context (cf. West and Pennell, 2003; Montgomery, 2020). A range of studies reported that underperforming students often showed lower levels of motivation, lower future  expectations, and more behavioral problems, compared to students who performed up to their expected level (Matthews and McBee, 2007; Mulder et al., 2007; Mercer and Pullen, 2009; Uno et al., 2010; Ziegler et al., 2012; Walkey et al., 2013). Underperformance in school was also observed to be negatively related to outcomes in later life. Underperforming students were at higher risk of dropping out of school and had lower wages and more health problems at later ages, compared to other students (Heckman and Rubinstein, 2001; Lan and Lanthier, 2003; Dianda, 2008).

In recent years, a range of social and emotional learning (SEL) programs were implemented in schools, targeted at the development of social–emotional skills among students, including those that underperform. These programs usually focused on self-awareness, self-management, social awareness, relationship skills, and responsible decision making, using the psychological theories mentioned before as guiding frameworks (Elias et al., 1997; Payton et al., 2000; Talvio and Lonka, 2013; Weissberg et al., 2015). There is an ongoing debate on whether these in-school programs are targeted at the right skills and whether it is at all possible to train social–emotional skills. Shriver and Weissberg (2020) recently provided an overview of the criticism. Students naturally have different dispositions in social and emotional skills. These skills are also shown to be variable and evolve over the life cycle as people age and (changes to) the environment influences the development of social and emotional skills. Childhood and adolescence are key periods of adolescent development. The magnitude of demands on social, regulatory, emotional, and moral capacities of children aged 6 to 18 years leads to pronounced changes in a number of their personality characteristics. This clearly demonstrates that personality is malleable during this period (Chernyshenko et al., 2018).

Whereas there is some general consensus that SEL programs should be targeted at intrapersonal and interpersonal skills and attitudes (Blyth et al., 2019), in-school programs were questioned on whether they targeted the right type of social–emotional skills among adolescents. Whitehurst (2019), for example, noted that some of the existing programs are too much focused on the development of abstract personality traits such as conscientiousness and should be more focused on specific skills, in line with cognitive development theory, which are directly linked to classroom practices. Another set of concerns was raised about the perceived role of using SEL programs as a “hyped” solution to more deeply rooted problems among adolescents such as violence and racism, but also the achievement gap between groups of students. It was stressed that although evidence showed positive effects on school performance in general, more empirical evidence was necessary to see whether SEL programs could be effective for specific problems or specific target groups, and more research was necessary to see how the development of social–emotional skills can best be assessed and monitored.

Several meta-analyses have examined the impact of school-based interventions to enhance SEL. For example, Durlak et al. (2011) performed a meta-analysis of 213 school-based SEL programs involving more than 270,000 students in primary and secondary schools. They found a moderately high standardized effect size showing that these programs can be effective. Other studies such as Martin (2005) showed that school motivation and school engagement of students could be improved by means of active workshops targeted at students’ planning, task management, persistence, self-efficacy, disengagement, valuing, mastery orientation, failure avoidance, and uncertainty control. By means of a randomized experiment where underperforming students in the treatment group received special sports activities targeted to boost their self-confidence and motivation, Heller et al. (2013) showed that such a program improved schooling outcomes. They observed a 0.14 standard deviation increase in an index comprising absenteeism, grades, and participation in the program during the intervention period. The Seven Habits of Highly Effective Teens, developed by Covey (2002), demonstrated the importance of certain habits among students for school performance, such as having a proactive attitude toward studying, prioritizing, goal orientation, and being able to respond to and manage changes in life (Prevoo, 2013).

In addition, positive effects were observed in the program, Lions Quest Skills for Adolescence (Laird and Roden, 1991; Laird et al., 1998; Eisen et al., 2003; Talvio and Lonka, 2013; Gol-Guven, 2016). This program was originally targeted to help students cope with difficulties in their lives, such as to prevent or free them from using drugs or violence, and developed into a more general SEL program in schools (Talvio and Lonka, 2013). The program aimed, among other things, to teach students cognitive–behavioral skills for building self-esteem and personal responsibility, communicating effectively, making better decisions, and resisting social influences among adolescents. It was designed for school-wide as well as classroom implementation in grades 6 to 8. Evaluation studies, using group-randomized trials, showed that the program led to higher self-esteem and assertiveness among girls, lower absenteeism during and after the intervention period, and on average an increase in students’ grade point average (GPA), from 2.1 to 2.3 on a scale from 0 to 4 (Laird and Roden, 1991; Laird et al., 1998; Bauer, 2004). These studies indicate that systematic interventions can change social and emotional skills of students in a desired direction and that these programs can be effective.

Most of these experimental studies were not specifically targeted at underperforming students, but at the entire student population. Results might be driven by the students who do not underperform. Because lack of motivation is commonly associated with underperformance, a challenge for interventions targeted at underperforming students is to keep students involved in the activities of the intervention. The question is whether those students who could benefit the most from a program targeted at social–emotional skills have a higher likelihood of dropping out of the program and whether observed effects of the program differ between those students who have most to gain and the others.

### The Current Study: Defining Underperformance

The current study focuses on underperforming students in secondary education. No standardized definition of underperformance has been used in the literature or in educational policy or practice. The concept might have different connotations to different persons, and it is not always clear what kind of definition or measurement is used. This might complicate the debate on underperformance. In general, underperformance refers to a discrepancy between a student’s (expected) performance potential and his/her actual or observed school performance (Smith, 2003; Phillipson, 2008; Veas et al., 2016). In the literature, underperformance was defined both on the individual and on the group level. Most studies using the individual-level definition of underperformance focused on gifted students, where it was commonly referred to as underachievement, yet some studies focused on the non-gifted as well (Phillipson, 2008). In such studies, either IQ tests or achievement tests were used to define the expected performance potential (Reis and McCoach, 2000). In other studies, underperformance was defined in terms of groups of students underperforming in relation to other groups, for example, boys versus girls (Burns and Bracey, 2001; Myhill, 2002; Watson et al., 2010; Bertrand and Pan, 2013), or students from lower socioeconomic backgrounds versus those from higher socioeconomic backgrounds (cf. Croizet and Claire, 1998), or differences between various ethnic groups in a country (Reisel, 2011).

Some studies on students’ school performance in Netherlands reported that both performance and school motivation of Dutch secondary school students were inadequate (Onderwijsraad, 2007; OECD, 2016). In line with these findings, teachers from Dutch secondary schools expressed their concern to us about underperformance of students especially in the early years of secondary school, in relation to low school motivation and engagement, and a lack of self-concept of their ability. Several studies showed that the transition from primary to secondary school was associated with an increased cognitive demand of students, as students were confronted with a larger variety of subjects and teachers, a higher difficulty of the content to be learned, deadlines and homework, and more normative and more frequent types of assessment (e.g., Anderman, 2013). Studies also showed that this transition was likely associated with a decline in motivation, achievement, and school engagement (Anderman, 2013; Martin, 2009, 2015). In our conversations with the teachers, we talked about what they meant with underperformance among their students, and we learned that they seemed to mix the two types of definitions given above. They compared a student’s performance to that of others in class (i.e., the group comparison), yet they also compared it to the expectations they themselves had of the student (i.e., individual-level). Research showed that teacher expectations commonly included not only expectations derived from observed performance, for example, by using achievement tests, but also more subjective expectations based on beliefs, stereotypes, or prejudices (cf. Brophy, 1983; Good, 1987; Weinstein, 2002; Babad, 2009; Rubie-Davies, 2010).

The Dutch teachers frequently mentioned things, such as “I expected more of this student, given the performance (s)he showed on the exit test in primary education. We know (s)he can do it, but (s)he does not show it.” In the Dutch education system, students are tracked when they move from primary to secondary school^1^. At the time of the current study, an exit test was used as the main determinant for track allocation. It was supplemented with a more subjective recommendation from the teacher, but this was conditional upon the exit test score. The impact of the exit test score on expectations about students’ school performance in secondary school was large, not only from teachers but also from parents and students themselves. To some extent, this was also driven by the fact that one of the quality indicators for secondary schools was whether students in ninth grade were still on the level of their track recommendation (Inspectorate of Education, 2017). In the operationalization of our underperformance measure, we derived students’ learning potential or expected performance from the exit test at the end of primary education, that is, in terms of achievement abilities. This expected performance was compared with both objective performance indicators in ninth grade, and with subjective performance indicators from teachers and parents.

### The Current Study: A Field Intervention in Education in a Research–Practice Partnership

In order to raise the performance of underperforming students, some Dutch teachers already experimented with changes in their instruction methods. They either used their own ideas, or they were also inspired by programs they had heard or read about, such as the abovementioned Lions Quest and the Seven Habits of Highly Effective Teens. They were curious to find out whether the use of these programs would prove to be effective when using proper research methods, that is, beyond the positive effects they experienced in class. They approached us as researchers to help design a classroom intervention and add to our knowledge on whether and how educational interventions could foster the development of social–emotional skills. This fits with the growing demand for evidence-based education and the use of field experiments in schools that support more ecologically valid causal analyses, compared to laboratory experiments (cf. Brown, 2015; Brown et al., 2017). Some studies have shown that experiments in schools that were targeted at improving academic achievement were mostly research-oriented; that is, they involved a lot of support by, or even depended on, researchers (Dignath and Büttner, 2008; Levin, 2013; Paunesku et al., 2015). This could raise difficulties when the intervention must be transferred to school practice by teachers that might not understand all the important features of the interventions, or in schools with different environments that do not fit the design of the intervention (Borghans et al., 2016). Designing an intervention together with schools minimizes application problems in practice and increases scalability (De Wolf and Borghans, 2012). However, designing interventions in co-creation between research and educational practice, that is, in research–practice partnerships, is complex. Consensus must be found between scientific rigor and practical relevance and applicability (cf. Penuel and Gallagher, 2017; Destin, 2018). It is not always feasible to use standardized research designs in educational practice, because every classroom is run differently, and the research design must allow for this variation. In addition, often a compromise must be made between the use of standardized scientific measures and measures available in educational practice.

As shown in the literature above, underperforming students could have problems in multiple domains of social–emotional skills. We asked the teachers to choose the most important domains, as we wanted to connect the intervention to the classroom problems they struggled with. In the end, the program was expected to become part of the curriculum, if proven effective. As a result, targeted outcomes of the current intervention are school performance (GPA), school engagement (school motivation and hours spent on homework), and self-concept (of school tasks and leadership skills). The chosen assignments of the intervention were aimed at raising students’ self-awareness about their school attitude and study behavior, and at encouraging them to think about future goals and aspirations.

Teachers impacted the choice of domains to include in the intervention and which outcomes to focus on. However, the researchers defined other elements of the research design. For example, it was stressed that randomization of treatment and control groups was necessary to establish (reliable) effects and circumvent any selection biases. For the final operationalization of the measures used in the intervention, that is, measures to establish the target group of students or to assess the outcomes of the program, both teacher experiences and researchers’ demands were balanced. To ensure the scientific validity of the field intervention, first a pilot study was executed to test and further shape the design of the treatment. Second, the design of the intervention, including the pilot study and the measures, was judged by a scientific committee. This approval was a prerequisite to receive funding for the intervention. The details of the intervention are explained in the following section.

Following the expectancy-value models and self-regulatory mechanisms explained before, the idea for the intervention program was that when students generally have a better idea of how they study, they are more able to organize their study tasks; they are more able to define realistic goals in advance; they are more able to monitor their progress; and they are more likely to be motivated to perform, persist when they encounter difficulties, have more realistic beliefs about their own capabilities, and in the end perform better. This is likely to be especially beneficial for underperforming students, as they more often have difficulties in these domains. Therefore, we investigated the following research question: To what extent does an in-school program aimed at students’ self-reflection on their study behavior, the organization, planning and monitoring of their study tasks, and the formulation of more realistic study goals affect GPA, school engagement and social and academic self-concept of underperforming students in secondary education?

## Materials and Methods

### Participants

#### The Sample of Schools and Tracks

The intervention targeted students in 9th and 10th grade (i.e., approximately age 15–16 years) who attended either the theoretical stream of the pre-vocational education track or the pre-higher education track in secondary education^1^. These two tracks were selected for the intervention, as underperformance and low student motivation were most common in these two tracks (Onderwijsraad, 2007). Students in these tracks are generally concentrated in the middle of the overall ability distribution and comprise a more heterogeneous group in terms of performance than students in the lowest and highest tracks. Earlier studies showed that some behavioral and performance-related problems were likely related to the transition from primary to secondary school (Driessen et al., 2005; Anderman, 2013; Martin, 2009, 2015). Participants were selected in ninth grade as transition-specific problems were expected to have disappeared within the first 2 years of secondary school.

To determine the number of students needed for the intervention, a power analysis was conducted^2^. This power analysis showed that a sample size of 200 students was sufficient to find an effect of 0.4-point increase in students’ GPA. The schools were recruited from secondary schools that were part of an ongoing regional research–practice partnership. This partnership included Maastricht University, primary and secondary schools, schools for vocational and higher education, and government bodies in the south of Netherlands (the Educatieve Agenda Limburg)^3^. This partnership supports schools in evidence-informed decision-making, whereby strong collaboration between educational research and practice and a regional monitor are key ingredients. Since 2010, approximately 90% of Dutch secondary schools in the region have been involved in the regional monitor. The 2012 cohort, from which we selected students, included 28 schools offering pre-vocational education (with 2,406 students) and 25 schools offering pre-higher education (with 2,405 students). Eighteen secondary schools participated in the intervention study: 10 pre-vocational education schools (with 992 students) and 8 pre-higher education schools (with 901 students). The regional monitor provided basic information about the non-participating schools as well, which allowed a check to what extent schools that participated in the intervention study constituted a selective sample. This is followed up in the *Discussion*. Additionally, the regional monitor provided information on students’ test scores that were used for the target group selection procedure, which is explained below.

#### Defining the Target Group of Underperforming Students

The selection of students for the intervention was aimed at students who showed underperformance in ninth grade in relation to their expected performance based on their primary school exit test score. This definition of underperformance is therefore based on achievement ability. The target group of underperforming students was determined using a two-step approach. In step 1, objective test scores from primary and secondary school were combined to determine the discrepancy between expected and observed performance. To validate this selection process, in step 2 additional subjective information from teachers and parents on students’ school performance was used. This procedure was also used in previous studies (e.g., Lavy and Schlosser, 2005; Holmlund and Silva, 2014). Feron et al. (2016) showed that the information provided by teachers complemented the assessment of students’ ability through standardized tests.

For step 1, we needed an objective measure for students’ school performance in ninth grade. Grades were not collected for the regional monitor and were generally not comparable across schools. Nor was a standardized test available to compare students from different schools, so a test was developed for the regional monitor. This was done in close cooperation with ninth-grade teachers and according to them served as a good proxy for the observed school performance of their students^4^. The reliability score (EAP) of the ninth-grade test score was 0.78 in both tracks. For 13% of the students at the participating schools in this study, no test data were available. These students were likely absent on the test day or completed too few questions on the test for a reliable score to be calculated, or their parents did not give consent for them to participate in the regional monitor. Information about the school performance for this group of students was gathered in step 2 of our approach.

To derive students’ expected performance, their primary school exit test scores, that is, in sixth grade, were used. This exit test score was suitable to test a student’s performance potential, because it was used for the track recommendation for secondary school (Feron et al., 2016). Therefore, students were expected to show their maximum performance^5^. The reliability score (KR20) for the 2009 exit test was 0.91 (CITO, 2009).

**FIGURE 1:**
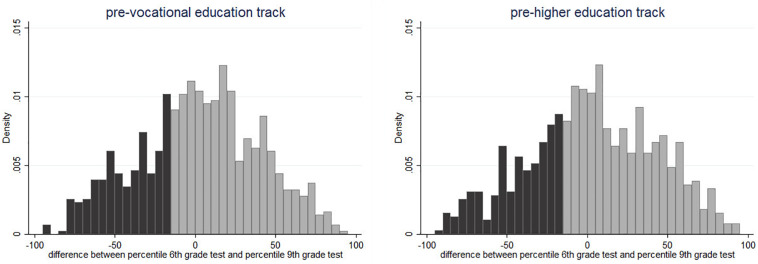
Difference in relative position in test scores between ninth and sixth grade. The figure shows the difference between the percentile in which the student was in the ninth-grade test (current performance) and the percentile in which the student was in the sixth-grade test (expected performance). The dark-colored group is the 25% of students who were selected for the intervention.

Table 1 shows descriptive statistics of the test scores and the number of students in the participating schools. For 861 students in the pre-vocational education track and 779 students in the pre-higher education track, information on both tests was available. To finish step 1 of our selection method and to determine the discrepancy between the students’ expected and observed school performance, both scores were divided in percentiles, by school and by study track. The percentile groups were composed at the school (and track) level, because when assessing students’ performance, teachers tended to compare students to their peers, as explained before. In most schools in the sample, students from different classes within the same track had the same teacher for a given subject. Accordingly, we did not compose the percentile groups at the classroom level. Finally, the difference between the two percentile distributions was calculated for all students. A negative difference implies that students had a higher relative position in the sixth-grade test compared to the ninth-grade test. Figure 1 shows the distribution of these percentile differences for students in both tracks.

**TABLE 1:** Number of observations (N), means (M) and standard deviations (SD) of tests used to define the target group for the intervention.

	Pre-vocational education track	Pre-higher education track
Students at participating schools		
N	992	901
Ninth-grade test score^1^		
N	861	779
Mean	0.48	0.53
SD	0.18	0.15
Sixth-grade test score^1^		
N	861	779
Mean	0.57	0.74
SD	0.15	0.13

*^1^Test scores are put on a 0- to 1-point scale. The ninth-grade test score is a sum score. The sixth-grade test score is determined by Cito and ranges from 500 to 550 but is transferred to a 0- to 1-point scale here. Section A3 of the Supplementary Material provides more details on the tests used.*

For the intervention study, students who were among the 25% of those who showed the largest discrepancy in percentiles between the two tests (i.e., lowest quartile) were selected as underperformers. The 25% cutoff was chosen to target a relatively broad group of underperforming students and to have enough power for the analyses. Moreover, some schools preferred to participate in the experiment only if they knew that at least a certain number of students were able to participate. Step 1 of the selection procedure resulted in a sample of 421 students (220 students in the pre-vocational education track and 201 students in the pre-higher education track).

Step 2 of the selection procedure aimed to validate the selection process of step 1, using additional subjective information from teachers and parents on the students’ school performance. The full list of selected students was discussed with teachers who served as mentor in ninth grade. Parents were also involved in the validation process. At all schools, information evenings were held, where parents were informed about the details of the intervention, and beforehand they were informed whether their child was considered to be an underperforming student or not. In some cases, teachers and parents argued that certain students should not be eligible for treatment, because their observed underperformance was only of a temporary nature or because of personal circumstances. In addition, teachers and parents added other students to the sample that did not emerge from the first step of the selection procedure. These were mainly students for whom no achievement test scores were available in step 1. In total, 363 students were identified for the final sample population (209 students in the pre-vocational education track and 154 students in the pre-higher education track). According to power analysis, this was sufficient to perform the analyses. Whereas this group of students might not necessarily be recognized as underperformers when using conventional methods, they were students who could do better in class, considered by their teachers and parents, and shown by test scores.

Within each educational track, schools were randomly assigned to the treatment or the control group, resulting in five treatment schools and five control schools for the pre-vocational education track and four treatment schools and four control schools for the pre-higher education track. Randomization at the school level was chosen because students within one school and educational track are likely to be in contact with each other, which might lead to spillover effects between treated and non-treated students. Between-school contacts were less likely in our sample, because the schools were located in different cities. The randomized assignment to treatment or control group was performed by Netherlands Bureau for Economic Policy Analysis as an external party. In total, 202 students were in the treatment group, and 161 students were in the control group.

### Intervention Design and Procedures

#### Pilot Study

A small pilot study was executed before the start of the actual experiment. The pilot study was held at two schools. The pilot was intended to test specific aspects of the intervention design, such as the selection method, the appropriateness of the assignments, and the feasibility of the intervention in schools. The intention of the pilot study was not to complete the full treatment; thus, no treatment effects were measured. At the end of the pilot phase, two feedback rounds were organized. One feedback round was held with the students who participated in the pilot, and the other feedback round was organized with the teachers.

Several lessons were learned from the pilot study. First, we learned that the procedure we used for selecting the underperforming students worked. Teachers and parents agreed with the selected list of students, and even students themselves argued that they could do better in class. Second, the pilot showed that designing an experiment in co-creation with teachers resulted in teachers’ better understanding of the experimental design and created more willingness for them to participate. The teachers from the pilot study also helped to explain the design of the study to teachers from other schools in the actual intervention. Third, intensive communication with teachers appeared crucial for the proper execution of the intervention. This influenced the logistic feasibility of the intervention and the accuracy of the effect measurement. Finally, the pilot study contributed to the creation of the assignments within the intervention. Parts of the content, as well as the language used in the assignments, were adjusted based on the feedback we received from students and teachers.

#### Actual Intervention

**FIGURE 2:**
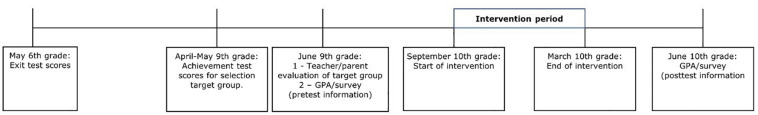
Timeline of the intervention.

The selection of students for the intervention took place in 9th grade, and the intervention was executed in 10th grade. Figure 2 shows the intervention timeline. During the intervention period, students in the treatment group were offered seven monthly assignments. These assignments aimed at raising students’ self-awareness about their school attitude and study behavior and encouraging them to think about future goals and aspirations. The assignments were motivated by the psychological theories explained in the introduction and the existing SEL programs of the Lions Quest and Seven Habits of Highly Effective Teens. The assignments were adapted to the Dutch school context by teachers and students in the pilot study^6^. The assignments were completed online and supervised by the responsible teacher or mentor. Students also had to reflect on the assignments with their teacher. The treatment took part during school hours, either in hours in which no classes were scheduled (so-called study hours at school within the curriculum), or in hours devoted to time with the mentor. We argued that this was likely to increase participation in the treatment, because it ensured that students received the treatment in a known and fostering learning environment and allowed them to ask questions. Teachers could remind students to participate in the intervention, but they did not force them to complete the assignments. They believed that forcing unwilling students did not contribute to their school motivation and their school performance. To prevent students from dropping out of the program, a small monetary incentive was used. Students in the treatment group who completed at least five of the seven assignments in the intervention received 25 Euros for their participation. They were told so at the start of the program. Students in the control groups did not receive any treatment (i.e., they did not complete any of these assignments); they followed their regular curricular courses.

Before and after the intervention period, students in both the treatment and the control group completed a survey. The pre-intervention survey was part of the regional monitor. While this limited the questions that could be chosen for measurement of the concepts that we were interested in, it enabled the use of questions that previously had been used for students in this age group, in a regular school context. For the schools’ participation in the intervention, it was important to not conduct an additional survey, as schools were overwhelmed with the number of (research) surveys. The post-intervention survey was taken at the very end, so the information from this survey was available only for those students who completed the full treatment. Consequently, for some outcome variables, the number of observations was too low to assess reliable effects of the intervention.

### Measures

Three types of outcome measures were used to evaluate the intervention: (1) students’ compliance with the treatment, (2) students’ GPA, and (3) students’ school engagement and self-concept in school tasks and leadership skills.

#### Completion of Treatment

We first examined which students completed the treatment and which students did not. Because underperforming students were the target of the intervention, this was a relevant outcome variable. Completion of the treatment was potentially related to motivational attitudes and could inform us whether interventions on social–emotional factors related to students’ school attitude, future goals, and aspirations were likely to succeed. We defined students as treatment group compliers when they completed at least four of the seven assignments. This meant that they received just over half of the treatment. As a robustness check, we also estimated all models using different definitions of treatment compliance, that is, ranging from completing one to all seven assignments.

Demographic control variables included in the analysis of treatment compliance included the educational track that students attended (pre-vocational education track or pre-higher education track), students’ age measured in months, gender, parental education, and region of birth. Parental education was measured by taking the education level of the highest educated parent, and we distinguished between (1) primary or lower secondary education, (2) upper secondary education or lower tertiary education, (3) higher tertiary vocational education or higher tertiary academic education, and (4) unknown. Region of birth distinguished between (1) Netherlands, (2) outside Netherlands, and (3) unknown. For the last two variables, the categories “unknown” were included to keep as many respondents in the sample as possible.

#### Students’ GPA

In Dutch secondary schools, students’ performance is graded by individual teachers of all subjects, by means of tests that they administer during the year. These are not standardized tests, except for the final examination at the end of secondary school. The grades students receive are measured on a scale ranging from 1 to 10, where 1 represents the lowest and 10 the highest grade. Students receive an official report card that lists the average grades they obtained for all of the subjects they take. They need a sufficient overall GPA to be able to transfer to the next grade (usually > 5.5). For the pre-vocational education track, subjects include Dutch, English, French, German, math, science, biology, economics, geography, history, and civics. For the pre-higher education track, subjects include Dutch, English, French, German, math, physics, chemistry, biology, economics, geography, and history. Not all students took all subjects (depending on which profile they took in school), and for this study, the GPA was calculated based on the subjects students took. The reliability (Cronbach’s α) of the GPA score was 0.85 for both educational tracks.

#### Students’ School Engagement

School engagement included two scales: school motivation and hours spent on homework. Students indicated whether they agree or disagree with some statements about their motivation to go to school and their attitude toward learning in general. For example, “I am motivated to continue learning,” or “As soon as I can, I quit school.” The statements were largely based on the Inventory of School Motivation, developed by McInerney and Sinclair (1991), and the Motivated Strategies for Learning Questionnaire developed by Pintrich et al. (1991). Each statement was measured on a 5-point scale ranging from “fully disagree” to “fully agree.”^7^ The reliability (Cronbach’s α) of the school motivation scale was 0.70. The overall score was calculated using confirmatory factor analysis (CFA) was used to calculate the overall score, using structural equation modelling [SEM, with full information maximum likelihood (FIML)]. The standardized factor loadings ranged from 0.48 to 0.73. The Comparative Fit Index (CFI) was 0.99, the χ^2^ [2 degrees of freedom (df)] was 11.31, and root mean square error of approximation (RMSEA) was 0.07.

The second aspect of school engagement included in the study was the average hours per week that students spent on homework. We included the average total hours spent on homework, that is, both at home and at school during study hours.

#### Students’ Self-Concept

We distinguished between two types of self-concept: school tasks and leadership skills. Following the work of Marsh (1992), students were asked to rate themselves on a range of skills used in school (e.g., arithmetic, writing) and on their behavior toward others (e.g., taking the lead). The skills were rated on a scale ranging from 1 to 10^8^. The reliabilities (Cronbach’s α) of the two factors were 0.70 for school tasks and 0.80 for leadership skills. The overall scores were calculated using CFA (SEM/FIML). Standardized factor loadings ranged from 0.41 to 0.71 for school tasks and from 0.59 to 0.77 for leadership skills. The model fit indices (CFI, χ^2^/df, RMSEA) for school tasks were 0.99, 5.66/2, and 0.05, and those for leadership skills were 0.99, 9.37/2, and 0.07.

### Statistical Analysis

Probit models were used to analyze students’ compliance with the treatment. The probit reflected the probability that a student completed at least four assignments. The probit models included the aforementioned demographic control variables. To facilitate interpretation of observed relations, marginal effects were reported.

Linear models of the treatment effect on student outcomes after the treatment were used to analyze the treatment effect on GPA, school engagement, and self-concept. These analyses included the levels of these outcomes before the treatment as lagged variables, or

$$Y_{i,t}=\beta_{0}+\beta_{1}\,D_{i}+\beta_{2}\,Y_{i,t-1}+\varepsilon_{i}$$

where *Y*_*i,t*_ represents the outcome variable in period *t* after the treatment, *D*_*i*_ equals 1 if the student was in the treatment group and equals 0 otherwise, *Y*_*i,t*__–__1_ represents the outcome variable in period *t* – 1 before the treatment, and ε_*i*_ represents the error term.

Three different ways of defining the treatment were used. The first definition used assignment to treatment (intention to treat or ITT): *D* = 1 for all students who were assigned to the treatment group at the start of the intervention. However, the treatment group non-compliers did not receive the full treatment. Therefore, a second definition used actual treatment participation: *D* = 1 for all students who completed at least four assignments. This model assumed that those who dropped out of the treatment also did not benefit from the assignment to treatment. If students self-selected into completing the actual treatment, or if continued participation was based on the expected gains from treatment, the conditional mean independence assumption is violated, and causal inferences are impossible. Such selection was plausible in our case. Therefore, a third definition used an instrumental variable approach, where assignment to the treatment was used as an instrument for the actual treatment taken (treatment effect on the treated or TOT). All treatment models included only students for whom both the pre-test and post-test variables were available. No imputations were made to the data.

We used standardized categorical outcome measures in all our models. The populations before and after treatment were no longer comparable because of the improvement of the treated population. Standardizing on the full population, that is, ignoring this, could lead to a biased estimate of the treatment effect, or in this case an underestimation of the effect size. Furthermore, as the observed dropout of the intervention was likely to be non-random, as explained before, an additional bias might be added to the estimates of the treatment effect. Consequently, we used the complying students in the control group as the basis for the standardization of variables in both outcomes [*cf.* Feron(2018, p221–222) for all details].

Finally, all models were estimated both with robust unclustered standard errors and with standard errors clustered at the school level, because observations might not be independent within schools. Moreover, the models that showed significant effects were also estimated with standard errors bootstrapped with clusters at the school level (400 reps), to see whether results held when simulating a larger sample of schools, because there were only 18 schools in the sample. All models were run in Stata/SE 14.0 (StataCorp, College Station, Texas, United States).

## Results

### Descriptive Statistics and Randomization Check

Table 2 provides descriptive statistics for students in the treatment and control groups, including some descriptive statistics for schools in the region that did not participate in the intervention. Schools representing the pre-vocational education track were somewhat overrepresented in the study. This has to be taken into consideration when generalizing the results. Table 2 also provides a comparison between the treatment and control groups as a check for successful randomization. Using bivariate *t* tests, no significant differences were observed between the treatment group and the control group on any of the observed student characteristics. A multivariate probit model confirmed this^9^. It was concluded that the randomization is successful.

**TABLE 2:** Number of observations (N), Means (M), standard deviations (SD), scale reliability (α), and model fit (CFI, χ^2^, RMSEA) for all measures.

	Schools in intervention						Schools outside intervention in region			Scale reliability	Model fit
	Treatment group			Control group							
					
	N	M	SD	N	M	SD	N	M	SD	α	CFI/χ^2^[df]/RMSEA
**Main variables**											
GPA t_0_	184	6.24	0.56	153	6.22	0.53	n.a.			0.85	
GPA t_1_	173	6.14	0.73	134	6.18	0.65					
Motivation t_0_^1^	161	–0.02	0.50	140	–0.08	0.60	596	0.03	0.52	0.70	0.99/11.31[2]/0.07
Motivation t_1_^1^	79	0.05	0.55	44	–0.00	0.54					
Hours homework t_0_^1^	151	6.30	4.41	139	5.55	3.85	572	5.89	3.70		
Hours homework t_1_^1^	79	7.36	4.38	44	8.42	7.08					
SC of school tasks t_0_^1^	153	–0.06	0.83	135	–0.04	0.93	550	0.03	0.73	0.70	0.99/5.66[2]/0.05
SC of school tasks t_1_^1^	79	0.03	0.93	43	–0.11	1.10					
SC of leadership skills t_0_^1^	154	–0.05	1.10	140	0.08	1.24	556	–0.01	1.05	0.80	0.99/9.37[2]/0.07
SC of leadership skills t_1_^1^	79	–0.71	1.49	43	0.00	1.46					
**Demographics t_0_**											
Share of students in pre-higher education track	202	0.42	0.49	161	0.43	0.50	703	0.52**	0.50		
Age in years	202	15.74	0.58	161	15.76	0.57	703	15.71	0.53		
Share of girls	202	0.43	0.50	161	0.36	0.48	703	0.45	0.50		
Parental education level	164	2.20	0.77	144	2.19	0.81	613	2.11	0.78		
Share of children born in Netherlands	169	0.96	0.20	150	0.96	0.20	624	0.98	0.15		

*t_0_ refers to pre-test information, t_1_ to post-test. At t_0_, differences were tested between treatment and control groups as well as between schools that participated in the intervention and those that did not. Differences at t_1_ are not tested here, because this is part of the treatment regressions. ^∗^*p* < 0.05, ^∗∗^*p* < 0.01, ^∗∗∗^*p* < 0.001. GPA, grade point average; SC, self-concept.^1^Standardized variables.*

Table 3 shows the bivariate correlations for all variables used in the analyses. Some interesting correlations were observed for GPA and school motivation. The results showed moderate correlations between GPA before and after treatment, and motivation before and after treatment. These correlations seemed stronger after the intervention than before. Similarly, the correlations between motivation and hours of homework seemed stronger after the intervention than before. These results could indicate that students with better grades were more motivated to participate in the intervention and spent more time on their homework following the intervention.

**TABLE 3:** Correlation among all variables in the analysis.

	1	2	3	4	5	6	7	8	9	10	11	12	13	14	15
1. GPA t_0_	1														
2. GPA t_1_	0.48***	1													
3. Motivation t_0_	0.19**	0.05	1												
4. Motivation t_1_	0.36***	0.28**	0.41***	1											
5. Hours homework t_0_	0.09	0.05	0.11**	–0.00	1										
6. Hours homework t_1_	0.07	–0.02	0.23*	0.22*	0.28**	1									
7. SC of school tasks t_0_	0.20***	0.06	0.20***	0.03	0.04	–0.00	1								
8. SC of school tasks t_1_	0.06	0.11	0.02	0.06	–0.09	–0.19	0.43***	1							
9. SC of leadership skills t_0_	0.02	0.05	0.07	0.07	–0.11	–0.02	0.54***	0.18	1						
10. SC of leadership skills t_1_	–0.09	0.09	–0.03	0.11	−0.24*	–0.06	0.11	0.16	0.55***	1					
11. Educational track	–0.09	−0.28***	–0.03	–0.00	0.06	0.15	0.02	–0.13	0.05	–0.06	1				
12. Age	–0.01	–0.08	–0.03	0.03	–0.07	–0.01	0.07	0.07	0.19**	0.26**	0.09	1			
13. Girl	0.09	–0.01	0.08*	0.11	0.07	0.07	–0.09	–0.12	–0.11	–0.12	–0.01	–0.10	1		
14. Parental education level	–0.02	0.04	0.05	0.07	−0.12*	0.08	–0.08	–0.16	0.04	0.08	–0.10	0.00	–0.03	1	
15. Born in NL	0.04	0.01	–0.06	–0.16	0.03	–0.05	–0.07	–0.05	–0.02	–0.11	–0.07	−0.13*	0.07	–0.05	1

t_0_ refers to pre-test information, t_1_ to post-test. *p < 0.05, **p < 0.01, ***p < 0.001. GPA, grade point average; SC, self-concept.

### Compliance With the Treatment

**FIGURE 3:**
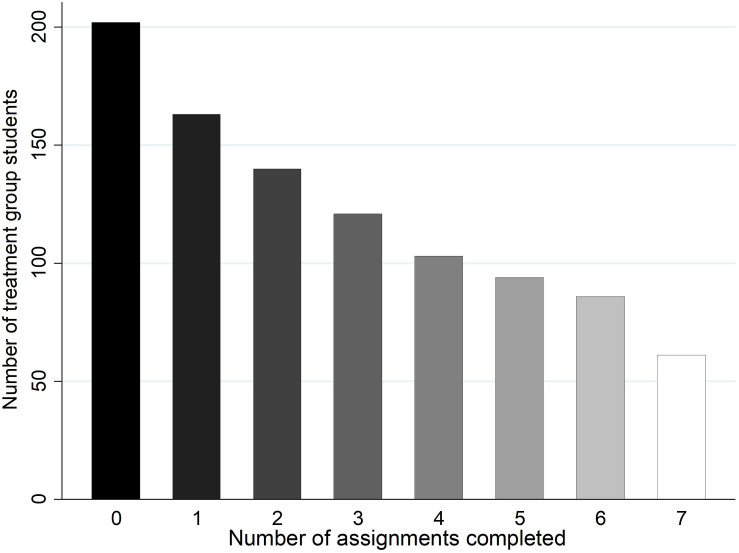
Compliance with treatment. The figure shows the number of students who completed the different assignments in the intervention program.

Figure 3 shows the number of treatment group compliers per assignment. Because of the nature of their school problems, that is, not performing up to their potential and low school engagement, the students in the target group had a relatively high probability of dropping out of the treatment. It proved indeed difficult to keep them involved in the program. We observed a gradual increase in the number of students who stopped completing the assignments during the intervention period; 51% of the students participated in at least four assignments.

Next, we examined to what extent compliers and non-compliers systematically differed from each other. Table 4 shows the results of the probability to complete at least four assignments for students in the treatment group. Model 1 in Table 4 included only GPA and controls and showed that older students were more likely to comply with the treatment. Model 2 showed similar results for students who completed the treatment. In model 3, measures for school motivation, homework, and self-concept were included. Students with higher pre-test school motivation were more likely to comply with the treatment, whereas students who showed a higher pre-test self-concept of school tasks were less likely to comply. A standard deviation increase in reported school motivation was related to a 29% increase in the likelihood to comply with the treatment. A standard deviation increase in self-concept of school tasks was related with a 17% decrease in the likelihood to comply with the treatment. These results remained significant when accounting for school fixed effects. The results remained borderline significant after bootstrapping the standard errors (with *p*-values of 0.067 for motivation and 0.059 for self-concept of school tasks), suggesting some weakness in the robustness of the relations. As a further robustness check, different models of compliance were estimated, ranging from completing one to seven assignments. The observed relations were significant in most models, but because of low numbers of observations, the models with compliance measured as completing over six assignments were statistically unstable.

**TABLE 4:** Marginal effects of probit models for compliance with the treatment.

	Model 1			Model 2			Model 3		
	β	*p*	95% CI	β	*p*	95% CI	β	*p*	95% CI
**Main variables (pre-test)^1^**									
GPA	0.01	0.892	[−0.13, 0.15]	–0.12	0.153	[−0.29, 0.05]	–0.18	0.061	[−0.35, 0.08]
Motivation							0.29***	0.009	[0.07, 0.50]
Homework (hours)							0.00	0.943	[−0.02, 0.02]
SC of school tasks							−0.17*	0.032	[−0.33, −0.01]
SC of leadership skills							0.09	0.104	[−0.02, 0.19]
**Demographic controls**									
Pre-higher education track	0.15	0.080	[−0.02, 0.31]	0.09	0.352	[−0.10, 0.27]	0.10	0.284	[−0.08, 0.29]
Female	–0.01	0.924	[−0.16, 0.15]	–0.07	0.451	[−0.25, 0.11]	–0.07	0.472	[−0.25, 0.12]
Age (in months)	−0.02***	0.001	[−0.03, −0.01]	−0.02**	0.005	[−0.03, −0.01]	−0.02**	0.001	[−0.04, −0.01]
Parental education: vocational	0.04	0.707	[−0.18, 0.27]	0.05	0.641	[0.17, 0.28]	0.02	0.892	[−0.23, 0.26]
Parental education: higher	0.18	0.092	[−0.03, 0.40]	0.18	0.102	[−0.04, 0.40]	0.14	0.237	[−0.09, 0.38]
Parental education: unknown	–0.07	0.639	[−0.38, 0.23]	–0.29	0.331	[−0.86, 0.29]	–0.34	0.200	[−0.87, 0.18]
Born in Netherlands	–0.12	0.525	[−0.47, 0.24]	–0.19	0.289	[−0.54, 0.16]	–0.20	0.310	[−0.58, 0.18]
Country of birth unknown^1^	0.14	0.523	[−0.28, 0.55]						
Average probability of treatment compliance	0.56			0.58			0.59		
Number of observations	184			136			136		

This table shows the marginal effects (dy/dx) from probit regressions where the dependent variable is equal to 1 if the student completed at least four assignments and 0 otherwise (students in the treatment group only). Y is the average probability of treatment compliance. Models were estimated using robust standard errors. Models with standard errors clustered at the school level and with bootstrapped standard errors showed no differences. *p < 0.05, **p < 0.01, ***p < 0.001. GPA, grade point average; SC, self-concept.^1^This category was used to keep as many students in the first model. In the later models, no information on the social–emotional variables was available for the students in this category and therefore is dropped from the study.

These results suggested that continued participation in the treatment was likely to be selective. Therefore, it was decided that estimating an ITT effect on outcome measures for these students would dilute the estimates of possible treatment effects, and estimating a TOT effect was likely to be more accurate.

### Treatment Effects on GPA and Social–Emotional Outcomes

Table 5 reports the estimated coefficients of the treatment on students’ GPA for all models. Model 1 used assignment to treatment as the treatment variable (ITT), model 2 used completion of at least four assignments as the treatment variable, and model 3 used assignment to the treatment group as an instrument for completion of at least four assignments (TOT). The results showed no treatment effect of the intervention on students’ GPA. Furthermore, it was observed that the treatment effect, where assignment to treatment was used as an instrument for treatment taken (model 3), was approximately twice the size of the ITT estimates of model 1. This is consistent with approximately 50% of the students not complying with the intervention. We also checked whether there were any heterogeneous treatment effects, as an overall null effect could be the result of contrasting results among groups of students^10^. No systematic heterogeneous treatment effects were observed.

**TABLE 5:** Treatment effect on GPA after treatment.

	Model 1			Model 2			Model 3		
	β	*p*	95% CI	β	*p*	95% CI	β	*p*	95% CI
Treatment	–0.05	0.435	[−0.18, 0.08]	–0.03	0.706	[−0.17, 0.11]	–0.09	0.433	[−0.33, 0.14]
GPA before treatment	0.66***	0.000	[0.48, 0.84]	0.66***	0.000	[0.48, 0.84]	0.66***	0.000	[0.48, 0.84]
Pre-higher education track	−0.38***	0.000	[−0.51, −0.24]	−0.38***	0.000	[−0.51, −0.24]	−0.37***	0.000	[−0.50, −0.24]
Constant	2.91***	0.000	[1.65, 4.16]	2.87***	0.000	[1.61, 4.14]	2.91***	0.000	[1.68, 4.14]
*R*^2^	0.303			0.302			0.298		

Model 1 used assignment to treatment as the treatment variable (intention-to-treat); model 2 used completion of at least four assignments as the treatment variable; and model 3 used assignment to the treatment group as an instrument for the treatment variable (treatment-on-treated). GPA before treatment and educational track were included as controls. Models were estimated using robust standard errors. Models with standard errors clustered at the school level and with bootstrapped standard errors showed no differences. *p < 0.05, **p < 0.01, ***p < 0.001. The number of observations was 294 in all models. GPA, grade point average.

In a final step, we analyzed whether the treatment had an effect on the students’ school engagement, including school motivation and hours spent on homework, and self-concept of school tasks and leadership skills. Table 6 shows that there were generally no observed effects from the treatment on these outcomes, except for a negative effect of the treatment on student’s self-concept of leadership skills. This result remained significant in models with standard errors clustered at the school level, or with bootstrapped standard errors.

**TABLE 6:** Treatment effect on school engagement and self-concept after treatment.

	School engagement						Self-concept on					
	Motivation			Hours spent on homework			School tasks			Leadership skills		
	β	*p*	95% CI	β	*p*	95% CI	β	*p*	95% CI	β	*p*	95% CI
Treatment	–0.05	0.622	[−0.14, 0.24]	–0.77	0.239	[−2.06, 0.52]	0.18	0.315	[−0.18, 0.54]	−0.63*	0.012	[−1.12, −0.14]
Dependent variable t_0_	0.41***	0.001	[0.18, 0.64]	0.24*	0.012	[0.05, 0.43]	0.50***	0.000	[0.33, 0.66]	0.70***	0.000	[0.44, 0.95]
Constant	−1.80**	0.005	[−3.05, −0.55]	3.14	0.401	[−4.24, 10.52]	0.13	0.910	[−2.12, 2.38]	1.39	0.340	[−1.49, 4.27]
*R*^2^	0.237			0.093			0.196			0.354		
Number of observations	108			104			103			106		

All models in this table were based on treatment defined as treatment completion of *all* assignments, as post-test information on school engagement and self-concept is not available for non-compliers. GPA before treatment is included as a control in all models (this predicts the outcome variables and thereby increases precision of the measurements for the treatment effect). Models were estimated using robust standard errors. Models with standard errors clustered at the school level and with bootstrapped standard errors showed no differences. *p < 0.05, **p < 0.01, ***p < 0.001.

## Discussion and Conclusion

Using a randomized field experiment, in this study we investigated whether an intervention using self-reflection on school behavior of underperforming secondary school students affected their GPA, school engagement, and some domains of self-concept. With this study, we contribute to the ongoing debate on whether in-school programs targeted at the development of social–emotional skills are effective, in particular for specific target groups such as underperforming students. In talks we had with teachers, they frequently mentioned that they struggled with engaging students who do not perform according to what teachers (or parents) expect from them. Psychological theories pointed to the importance of several social–emotional skills for engaging students in school and raising their school performance. These skills included students’ expectations, perceptions, or beliefs about their competences and the difficulty of the tasks they have to do at school, their coping strategies when experiencing learning difficulties or challenging tasks, their goal-setting behavior, and their reflective monitoring of progress (cf., Karoly, 1993; Mayer et al., 2004; Wigfield and Cambria, 2010). It was argued that when students have positive beliefs about their own capabilities in relation to school tasks and are able to set realistic achievement goals, they are more likely to be motivated to start with the task and to persist when they encounter difficulties. This would lead to better school performance. Self-reflection on their study behavior and their expectations could help to achieve this.

An important feature of the study was that the intervention was designed in co-creation with teachers. Such codesigned intervention studies in education are becoming more common, in response to the gap between educational interventions developed by scientists and the practical applicability by teachers (Penuel and Gallagher, 2017). When the question arises from educational practice, cooperation of teachers is more likely. The scientific input for the design enhances reliability of observed effects, and generalizability and scalability of the intervention. Developing the research question and designing the intervention together could be an effective approach to target a specific problem in educational practice (Borghans et al., 2016). An important aspect of the intervention in this study was the ease and limited costs with which it could be scaled up. The treatment for the students had a low time intensity and was provided through an online platform. The possible disadvantage of the low-time intensity of the intervention was that the time scheduled for the intervention was too short to observe any effects.

Previous studies showed positive effects of interventions on enhancing school performance and school engagement of students in secondary education. The majority of these interventions targeted the entire group of students in a class or school or focused at underperforming students in relation to giftedness. An important feature of the present study was the sole focus on underperforming students. We specifically focused on students in the later years of secondary school, as evidence pointed to problems in the early years of secondary education resulting from an increased cognitive demand in comparison to the primary school learning environment. Most of the problems related to the transition from primary to secondary school were resolved within 2 years, when students found their way in secondary school. However, for some students, problems were more persistent and put them at risk of early school dropout.

It could be questioned whether the schools that participated in the study were a random group of schools. It was possible that schools were more willing to participate in the intervention if they experienced problems with students’ motivation or school performance. Based on additional information about the schools that did not participate in the study, we found that schools offering the pre-vocational education track were somewhat overrepresented in the study. This could imply that underperformance was more common in this track. No differences on the observed student characteristics were observed. From this, we concluded that at least based on these observables the participating schools were not different from the non-participating schools.

There are different ways to define underperformance. This study chose to define the target group of students who “could perform better” by comparing students’ observed school performance in ninth grade with high-stakes test results from sixth grade. Both tests used similar domains on which the students were tested and were important for the school curriculum, that is, math and language. A discrepancy between ninth- and sixth-grade school performance could, however, be due to different reasons than motivational deficits. For example, instructions in primary and secondary school are known to be different and might relate to low school performance and school engagement for some groups of students (cf., Becker et al., 2012). Other factors that affect the performance discrepancy could be related to the onset of adolescence or changes in parental involvement (Hopwood et al., 2016). Moreover, the observed discrepancy could be driven by differences in test motivation. Whereas the sixth-grade test was high stakes for the students, the ninth-grade test was low stakes. It has been shown that test scores were generally higher when the stakes of the test increased (e.g., Angrist and Lavy, 2009; Segal, 2012; Simzar et al., 2015). These reasons for the observed discrepancy applied to all students in school, yet apparently not all students were affected in a similar way, and some students “underperform.” We argued that by using these two test scores, we had a suitable selection mechanism for students who do not show their full learning potential in secondary school. This was supported by the fact that both teachers and parents confirmed the selection of students. Whereas such personal judgments could also include biases (e.g., Muijs and Reynolds, 2015), using the information from both the objective and subjective instruments provided a valid instrument for the selection of the target group for this intervention. The selection procedure was tested in a small pilot study, and not only teachers and parents, but also students themselves, agreed that they were correctly identified as underperforming students.

Targeting a group of students with lower than expected school engagement and school performance might increase the risk of dropout during the intervention. The first question this article therefore addressed was whether those students who could potentially benefit the most from the intervention, that is, whether those with the lowest school engagement or lowest GPA, had a higher likelihood of dropping out of the intervention. We found that students with higher school motivation before the treatment were more likely to comply, and students with higher self-concept of school tasks (e.g., math, writing) were less likely to comply. The latter relation was partly supported by a marginally significant negative relation between GPA and treatment compliance. In conclusion, among the students who were selected for the intervention, those who potentially gained the most of the program in terms of outcomes in the domain of school motivation and self-concept were more likely to drop out of the intervention. This finding is particularly interesting as this latter group was exactly the group that the program was trying to reach. As described before, recent studies pointed to the malleability of social–emotional skills in school (Durlak et al., 2011; Chernyshenko et al., 2018). The majority of the programs studied were applied to all students in class, and the observed effects of increased social–emotional skills on academic performance and motivation could well be driven by already more advantaged groups in class. More insights were needed to establish the malleability of social–emotional skills for specific groups of students, such as underperforming students.

Our results indicate how difficult it is to reach the particular target group of underperforming students with an in-school intervention. Even though the intervention was designed in co-creation with teachers, this did not prevent students from dropping out of the intervention. Continued participation, however, varied between schools. In some schools, low dropout percentages (<10%) were observed, whereas at other schools, high dropout rates (>60%) were observed. Teachers had an important role in coaching and supervising the students with respect to the intervention. Durlak et al. (2011) also showed that teachers were able to effectively conduct SEL programs in school. Although we had quite intense contact with teachers in most of the schools, it might be that not all teachers were equally motivated. Motivation of teachers seemed to be an important factor in motivating students, and variation in teacher motivation to participate in the intervention could explain differences in the dropout rates between the schools. As discussed in Borghans et al. (2016), for students to be motivated to participate in an intervention, it is important that schools and teachers support the intervention and facilitate students to take part in the program. Our study could indicate that this is even more important for in-school training programs involving students who have motivational problems.

In the second part of the analyses, we investigated whether the intervention had an effect on students’ GPA, school engagement, or self-concept. No robust overall effects of the treatment on students’ GPA and social–emotional outcomes were observed, except for a negative effect on self-concept of leadership skills. This latter finding could be due to the fact that students had to reflect on their own capabilities and self-esteem in the intervention and became more modest on their leadership skills. We did observe that the target group of students had a higher self-concept of leadership skills than the other students. So, after the intervention, the target group is now closer to the level of self-concept reported by non-underperforming students. Kerr et al. (2003) had shown earlier that when students in early adolescence become more oriented toward each other, this might also go along with more feelings of insecurity. Without further investigation, we cannot say more on the mechanisms behind this. However, it is questionable whether it is a real effect, or a coincidental finding among a small sample size. In addition, although not robust, there was a weak indication for the treatment to be more effective in raising GPA for those in the pre-vocational education track. This is interesting to explore in more detail in future studies. It might have implications for increasing school performance among certain groups of students who are more at risk, such as those of lower socioeconomic backgrounds. Previous studies showed that low school performance more frequently occurred among students from lower socioeconomic backgrounds and that these students were more commonly found in lower educational tracks (e.g., Walkey et al., 2013).

There are multiple possible explanations for why this study did not find significant treatment effects on students’ GPA, school engagement, and self-concept. First, the intensity of the treatment, with one assignment per month, might have been too low to significantly increase the outcomes. Second, the selection of underperforming students might make it more difficult to observe treatment effects as these students are less likely to participate in an intervention. Moreover, as a large number of students drop out during the intervention, the sample size for treatment effect analyses decreased, and finding significant effect sizes becomes more difficult. Third, the difference in dropout rates between schools could indicate that motivating teachers for an intervention is important.

Apart from these more operational reasons for not finding an effect, reasons could also be related to the design of the intervention and the measures used. For example, the randomization of treatment and control group was conducted at the school level, rather than at the class level. However, 18 schools might be too few for randomization to balance all potential confounders. While we trust the randomization using tests for group differences, unobserved factors might still drive differences between treatment and control groups. Finally, even though the factors for school engagement and self-concept show good factor loadings and sufficient to good internal consistency, the model fit indices do not always show optimal fit. Whereas the CFIs were good for all models, the χ^2^/df and RMSEA were acceptable (e.g., RMSEA between 0.05 and 0.07). It is not uncommon that the model fit indices provide contrasting information (cf. Barrett, 2007). It should be noted, though, that RMSEA tends to inflate when there are low df’s, which is the case in our models, especially those with the acceptable (but not optimal) model fit (Kenny et al., 2014). It could still be possible that the items included in one factor pick up different dimensions of self-concept, yet the confirmatory factor analysis of self-concept proved the existence of two distinct factors, and with less than the current four items per factor, content validity is at stake. Addressing these issues might lead to more beneficial results of the intervention. However, it could well be that even in that case there might be no effect of this specific in-school training program on students’ school performance, school engagement, or self-concept for this specific group of underperforming students.

Hulleman and Cordray (2009) argued that field experiments often showed smaller effects of interventions than those taken in laboratories. Reasons were differences, both observed and unobserved, in the implementation of the intervention and the multitude of contextual factors that came into play when the intervention was administered in real life. While we regularly met with the teachers who supervised the intervention in class, we were not present when the students took the assignments. There was no strict control over the implementation process, which might have led to unwanted behavior during the hours that students worked on the assignments. Large sample sizes are often needed to overcome such problems and reach adequate treatment effects (Gelman and Carlin, 2014). This is not always possible in educational settings and poses a trade-off to the researchers. In the power analysis that we calculated before approaching the schools, we already included a proxy for non-compliance, taken from evidence on compliance in other, mostly laboratory, experiments. When designing a field experiment, we learned that the size of this proxy should be substantial, to avoid measurement problems due to low sample sizes.

Despite the fact that mainly null effects were observed in this study, which were not related to weak power, and given the fact that the intervention was thoroughly designed in co-creation with educational practice and tested in a pilot study, results are worth sharing and disseminating. Recently, concern has risen about publication bias and disregard of null findings in educational research, whereas these studies are informative for educational policy, practice, and research and add to the pool of evidence-based research in education (Chow and Ekholm, 2018). Jacob et al. (2019) showed that even large-scale (quasi-)experimental studies in education, which were designed and executed appropriately, often show weak or null effects. They further showed that even those studies have merit for educational practice and research, because it helps to reveal information about complex learning mechanisms among students. This study adds to our knowledge on whether educational interventions or training programs in school can foster students’ social–emotional skills, such as motivation or self-concepts of specific groups of students.

## Data Availability Statement

The datasets generated for this study are available on request to the corresponding author.

## Ethics Statement

Ethical review and approval was not required for the study on human participants in accordance with the local legislation and institutional requirements. Written informed consent to participate in this study was provided by the participants’ legal guardian/next of kin.

## Author Contributions

TS designed the intervention. EF administered the intervention at the schools and prepared the data. EF and TS analyzed and interpreted the data and wrote the manuscript. All authors read and approved the final manuscript.

## Conflict of Interest

The authors declare that the research was conducted in the absence of any commercial or financial relationships that could be construed as a potential conflict of interest.

## Footnotes

1 Section A1 of the Supplementary Material provides more information on the Dutch education system.

2 Section A2 of the Supplementary Material provides more information on the power analysis.

3 For more information see https://www.educatieveagendalimburg.nl/onderwijsmonitor-p/english.

4 Section A3 of the Supplementary Material provides more information on the 9th-grade test scores for the students in the participating and non-participating schools.

5 Section A3 of the Supplementary Material provides more details on the test content and shows some information on the mean test scores for the students in the participating and non-participating schools.

6 Section A4 of the Supplementary Material provides a detailed description of the assignments.

7 Section A5 of the Supplementary Material shows the items used for the motivational scale.

8 Section A5 of the Supplementary Material shows the items used for the two domains of self-concept.

9 Table A4 in the Supplementary Material section A6 provides the estimation results.

10 Section A7 in the Supplementary Material presents the findings.
