# Supplementary material for: A Randomized Field Experiment Using Self-Reflection on School Behavior to Help Students in Secondary School Reach Their Performance Potential
Source: Front Psychol. 2020 Jun 30;11:1356. doi: 10.3389/fpsyg.2020.01356 (PMC7339948; doi:10.3389/fpsyg.2020.01356)
Supplement: Supplementary file 1 [file Data_Sheet_1.DOCX]

**Supplementary material**

A1. The Dutch secondary education system

Figure A1 shows an overview of the Dutch education system. Primary education consists of eight years of which the ﬁrst two are spent in kindergarten. Most children start kindergarten at the age of 4, enter 1st grade at the age of 6, and ﬁnish primary school at the age of 12. Students are sorted into tracks when they enter secondary school in 7th grade. The Dutch secondary education system is hierarchically structured by ability and consists of three main tracks that differ in duration and qualification. The four-year pre-vocational education track (*vmbo*) qualifies children for vocational education, the five-year pre-higher education track (*havo*) qualifies children for higher (professional) education and the six-year pre-academic track (*vwo*) qualifies children for university. On average, 55 percent of the children end up in the pre-vocational track, 25 percent in the pre-higher education track and 20 percent in the pre-academic track. Within the pre-vocational track, two streams are available: a more practically oriented stream and a more theoretically oriented stream. About 45 percent of the students in the pre-vocational track take the practical stream, and 55 percent take the theoretical stream.

*Figure A1 | Dutch education system*


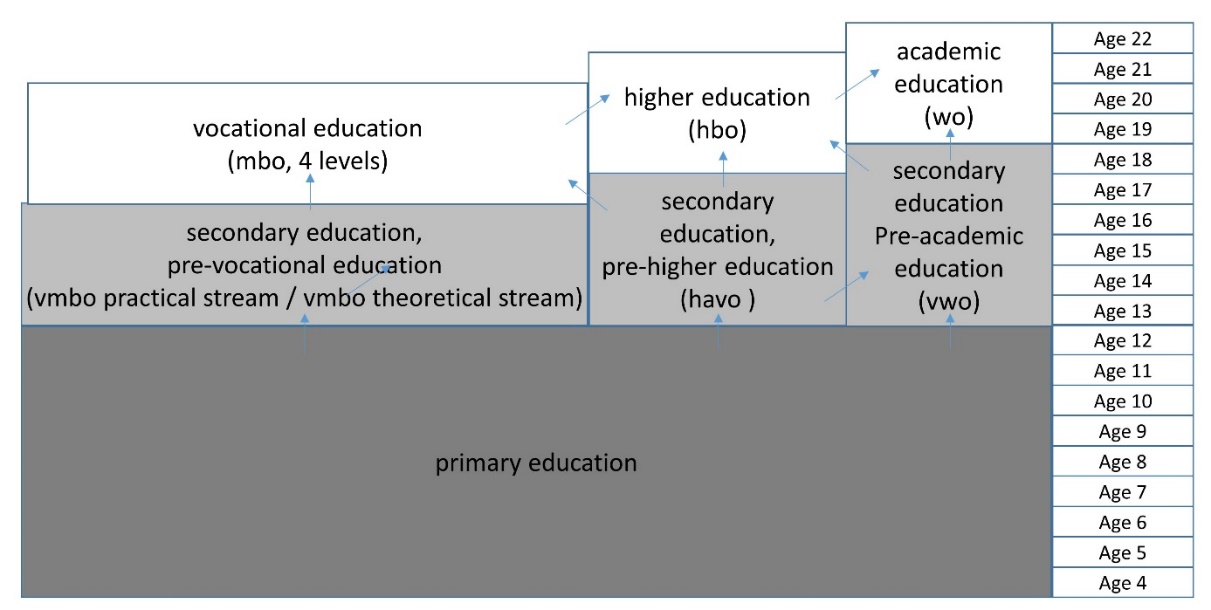


A2. Power analysis

A power analysis was conducted in STATA/SE 14.0, using GPA as the main outcome variable, and the expected effect size of the program as a main ingredient. A proxy for this latter was derived from the studies on the Lion’s quest program, and values for the expected effect size between 0.3 and 0.5 points increase in GPA were used in the power analysis. We also took into account some factors accounting for measurement error due to differences in GPA between students that are unrelated to the treatment, differences in grades between tests, and differences in grades between schools. Proxies for these measurement errors were obtained using existing data on students from the same region, of similar age, using similar measures used in the actual intervention study. Several models were estimated to calculate the power, including models with standard errors clustered at the school level. Feron (2018, p223-224) provides all details on the power analysis.

A3. Additional information on test scores used for defining underperformance

The (digital) test developed for the regional monitoring project to measure school performance of 9th-grade students included items from existing tests in the domains of language and mathematics. The language test contained items on comprehensive reading taken from the Program for International Student Assessment (PISA, OECD, 2009) and items on spelling and word knowledge taken from a Dutch Cohort Study (COOL5-18, Zijsling et al., 2009). The math test contained again items taken from PISA and COOL5–18, and some items from a Belgian Study on School Feedback (Verhaeghe & Van Damme, 2007). The exit test in 6th grade was developed and assessed by Cito, a testing company which is independent from the schools (van Boxtel et al., 2011). It was a three-day test, consisting of 60 questions on math, 96 on language, and 87 on world orientation (e.g. geography, biology and history). The content covered by this test was a bit wider than that of the 9th-grade achievement test.

Table A1 shows the mean scores (relative sum scores) for the students at the participating schools and those outside this study. Students in the pre-vocational education track at the participating schools showed somewhat lower test scores compared to the students in the non-participating schools. A t-test showed that this difference was significant (*t = 8.13*). The participating schools might have sorted themselves into the intervention study, because they have more problems with their students’ school performance. In the pre-higher education track, no differences were observed.

The table shows the same information for the scores on the exit test scores (conditional on that information on the 9th-grade test is available), and no differences were observed between the schools that participated in the intervention study and those that did not. The exit test score was not available for all students at the participating schools. This was the case for 102 students in the pre-vocational education track and 57 students in the pre-higher education track. Reasons for this were that the student attended a primary school outside the region, the primary schools that students attended did not participate in the regional partnership, or that the schools used an alternative exit test (85 percent of the school uses the test from Cito). For most of these students, we had an alternative objective test score. Within the regional monitoring project, 9th-grade students also completed an IQ-test. This test was taken from the beforementioned Dutch Cohort Study, and included IQ-questions in both the non-verbal, verbal and numerical domain (COOL5-18, see Zijsling et al., 2009 for details on the test). While this test was low stakes, in comparison to the exit test score from 6th grade, it is argued that an IQ-test could deliver a proxy of a student’s potential performance. For students for whom we had both the exit test score and the IQ-test score, Table A2 showed the percentages of students in similar parts of the distribution on both test scores. For about one-fifth of the students this position differed between the two tests. Given the fact that the two tests provided comparable results on a student’s position in the ability distribution, one could argue why we did not use the IQ-test anyhow as a basis for the students’ expected performance. As explained earlier, the performance of students on the exit test score was more commonly used as reference point for students in the Netherlands, and some studies also showed limitations of using IQ-tests to derive students’ learning potential (e.g. Hurks and Bakker, 2016).

*Table A1 | Descriptive statistics on test scores used to define underperformance*

|  | Pre-vocational education track | Pre-higher education track |
| --- | --- | --- |
| All schools in regional monitor: test score 9th grade^1^ | | |
| N  M  SD | 1,940  0.52  0.18 | 2,075  0.54  0.15 |
| Schools participating in the intervention study: test score 9th grade^1^ | | |
| N  M  SD | 863  0.48  0.18 | 779  0.53  0.15 |
| Schools participating in the regional monitor: exit test score 6th grade^2^ | | |
| N  M  SD | 1,701  533.5  5.8 | 1,819  539.65  4.7 |
| Schools participating in the intervention study: descriptives exit test score 6th grade^2^ | | |
| N  M  SD | 761  533.1  5.9 | 725  539.6  4.8 |

^1^Relative sum scores were used for this study. Using IRT-scores was not expected to lead to a different selection of students. Correlations between the sumscores and IRT-scores (2pl) were 0.92. ^2^This information is conditional on the sample of students for which the 9th-grade information is available. The exit test score is given on a scale of 500-550 and determined by CITO (CITO, 2009).

*Table A2 | Comparison of exit test score in 6th grade and iq-test in 9th grade*

| Exit test score 6th grade | | | | |
| --- | --- | --- | --- | --- |
| IQ-test score 9th grade | More than 1 SD below mean | Within 1 SD of mean | More than 1 SD above mean | Total |
| More than 1 SD below mean | 6.85 | 12.53 | .00 | 19.38 |
| Within 1 SD of mean | 9.49 | 32.38 | .00 | 41.87 |
| More than 1 SD above mean | .00 | .00 | 38.75 | 38.75 |
| Total | 16.34 | 44.91 | 38.75 | 100.00 |

A4. Assignments in the intervention study

The seven assignments had the following theme and content:

1. *Planning*. Students were asked to set up a weekly and monthly planning and set some goals. It was stressed that the planning should be realistic, include study time for tests, and allow for reflection time and ‘unexpected issues’. During the month, they had to keep track of the number of hours spent on certain tasks, and they had to reflect on their time-management and study behavior.
2. *Self-image*. Students were asked to write a short speech for their graduation in a few years. What would they like the teacher to say about them? This exercise was aimed to make them think about how they would like to be, what characteristics they would like to develop, and how they would like to be seen by others.
3. *Self-confidence*. Students were asked to explain what they think self-confidence constitutes, and how one could see whether someone is self-confident. They also had to think what advantages and disadvantages high or low self-confidence has. In a final step, they had to reflect on their own self-confidence.
4. *Skill creation*. Students were asked to reflect on the skills they think are important for later success in school, in a job and more general in life. They were also asked to what extent they think they master these skills and what they need they could develop them.
5. *Teamwork*. Students were asked to state their ideas on teamwork: what does it mean, when is it useful and when could it not be useful, what is needed to foster productive teamwork, and what factors can prevent productive teamwork. They were also asked to write down an example of productive team work from their own experience as well as an example where the teamwork was not so smooth. They had to identify the aspects that they thought were responsible for whether the teamwork was productive or not.
6. *Taking decisions and responsibility*. Students were asked to reflect on the extent they think they were responsible for their own decisions both in the short and the long run. They were asked what decisions they have to made in the near future and which factors affect how and what they decide.
7. *Expectations*. Students were asked to explain the influence others people’s opinion has on their behavior, to what extent they think they live up to their own or other’s expectations, and they had to give examples of good and bad influences. Furthermore, they were asked to describe a role model and specifically describe the characteristics that they value and aspire themselves.

A5. Supplementary information on measures used in this study

Table A3 gives an overview of the statements included in the measurement of school motivation and the four domains of self-concept.

*Table A3 | Items used for assessment of motivation and self-concept*

| *School appraisal / valuing:* | *Self-concept of school tasks*: | *Self-concept of leadership:* |
| --- | --- | --- |
| As soon as I can, I quit school (-)  I am motivated to continue learning  I am continuing to learn because I like it  I am continuing to learn for a long time | Mental arithmetic  Writing without mistakes  Following the news  Concentrating | Giving my own opinion  Getting what I want  Taking the lead  Debating |

A6 Supplementary material on randomization check

Table A4 shows the result of a multivariate analysis (probit models) for a comparison between the treatment and control group.

*Table A4 | Marginal effects of a probit model for being in the treatment versus the control group*

|  | β | p | 95% CI |
| --- | --- | --- | --- |
| *School performance, school engagement and self-concept^1^:* | | | |
| Average grade t_0_ | 0.03 | 0.634 | [-0.09, 0.15] |
| Motivation t_0_ | 0.09 | 0.132 | [-0.03, 0.22] |
| Homework (hours) t_0_ | 0.01 | 0.283 | [-0.01, 0.02] |
| Self-concept of school tasks t_0_ | -0.01 | 0.805 | [-0.10, 0.08] |
| Self-concept of leadership t_0_ | -0.01 | 0.740 | [-0.08, 0.05] |
| *Demographic variables*: | | | |
| Pre-higher education track | 0.05 | 0.478 | [-0.08, 0.17] |
| Female | 0.08 | 0.249 | [-0.05, 0.21] |
| Age (in months) | 0.00 | 0.851 | [-0.01, 0.01] |
| Parental education: vocational | 0.05 | 0.527 | [-0.12, 0.22] |
| Parental education: higher | -0.02 | 0.770 | [-0.19, 0.14] |
| Parental education: unknown | 0.09 | 0.659 | [-0.31, 0.49] |
| Born in the Netherlands | 0.07 | 0.665 | [-0.24, 0.37] |
|  |  |  |  |
| Average probability of being in treatment group | 0.52 | | |
| Number of observations | 260 | | |

This table shows the marginal effects (dy/dx) from probit regressions where the dependent variable is equal to 1 if the student was in the treatment group and 0 if he was in the control group. Y is the average probability of being in the treatment group. Models were estimated using robust standard errors. Models with standard errors clustered at the school level and with bootstrapped standard errors showed no differences. *p<0.05, **p<0.01, ***p<0.001. ^1^t_0_ refers to the value of the variable before treatment.

A7 Heterogenous treatment effects

Though the results did not show a significant overall effect of the treatment on students’ GPA, nor did we have strong theoretical priors on heterogeneity of effects among groups of students, it could well be that effects are nulled out to contrasting results among groups. Heterogeneous treatment effects were analyzed between boys and girls, between students from different educational tracks, between students with below and above mean GPA before the intervention, and between students with below and above mean school motivation before the intervention. Table A5 showed no systematic differences between these groups of students. There seemed to be a weakly significant (p<0.10) difference by educational track, with a lower treatment effect for those in the pre-higher education track, yet this finding was neither strong nor robust.

*Table A5 | Heterogeneous treatment effect on GPA after treatment*

|  | Model 1 | | | Model 2 | | | | Model 3 | | |  |
| --- | --- | --- | --- | --- | --- | --- | --- | --- | --- | --- | --- |
|  | β | p | 95% CI | β | p | 95% CI | β | | p | 95% CI |  |
| *Interaction with gender*: | | | | | | | | | | | |
| Treatment boys | -0.03 | 0.785 | [-0.21, 0.16] | -0.03 | 0.786 | [-0.25, 0.19] | -0.05 | | 0.785 | [-0.39, 0.30] |  |
| Treatment girls | -0.06 | 0.677 | [-0.32, 0.21] | 0.02 | 0.910 | [-0.27, 0.30] | -0.09 | | 0.705 | [-0.56, 0.38] |  |
| Girls | -0.01 | 0.941 | [-0.19, 0.18] | -0.05 | 0.569 | [-0.21, 0.11] | -0.01 | | 0.945 | [-0.19, 0.18] |  |
| Constant | 2.89^***^ | 0.000 | [1.64, 4.14] | 2.88^***^ | 0.000 | [1.59, 4.17] | 2.89^***^ | | 0.000 | [1.66, 4.12] |  |
| R squared | 0.304 | | | 0.303 | | | | 0.299 | | |  |
| *Interaction with educational track*: | | | | | | | | | | | |
| Treatment pre-vocational track | 0.53 | 0.103 | [-.011, 1.18] | 0.18 | 0.614 | [-0.51, 0.86] | 0.19 | | 0.640 | [-0.61, 1.00] |  |
| Treatment pre higher education track | -0.24 | 0.075 | [-0.51, 0.02] | -0.08 | 0.569 | [-0.37, 0.20] | -0.05 | | 0.491 | [-0.19, 0.09] |  |
| Higher education track | -0.24^**^ | 0.011 | [-0.43, -0.06] | -0.35^***^ | 0.000 | [-0.52, -0.18 | -0.34^***^ | | 0.000 | [-0.50, -0.17] |  |
| Constant | 2.55^***^ | 0.000 | [1.30, 3.80] | 2.83^***^ | 0.000 | [1.52, 4.12] | 2.84^***^ | | 0.000 | [1.59, 4.08] |  |
| R squared | 0.310 | | | 0.303 | | | | 0.299 | | |  |
| *Interaction with having low pre-test GPA:* | | | | | | | | | | | |
| Treatment above mean GPA | -0.11 | 0.210 | [-0.27, 0.06] | 0.06 | 0.559 | [-0.14, 0.26] | -0.22 | | 0.216 | [-0.57, 0.13] |  |
| Treatment below mean GPA | 0.13 | 0.334 | [-0.13, 0.39] | -0.17 | 0.249 | [-0.46, 0.12] | 0.26 | | 0.284 | [-0.21, 0.73] |  |
| Below mean GPA | -0.10 | 0.446 | [-0.36, 0.16] | 0.01 | 0.910 | [-0.22, 0.24] | -0.10 | | 0.464 | [-0.36, 0.16] |  |
| Constant | 3.04^**^ | 0.003 | [1.03, 5.04] | 3.09^**^ | 0.003 | [1.08, 5.10] | 3.00^**^ | | 0.004 | [0.98, 5.01] |  |
| R squared | 0.305 | | | 0.305 | | | 0.283 | | | |  |
| *Interaction with having low pre-test school motivation:* | | | | | | | | | | | |
| Treatment above mean motivation | -0.03 | 0.734 | [-0.22, 0.15] | 0.02 | 0.850 | [-0.17, 0.20] | -0.05 | | 0.723 | [-0.33, 0.23] |  |
| Treatment below mean motivation | -0.04 | 0.770 | [-0.29, 0.22] | -0.11 | 0.484 | [-0.41, 0.20] | -0.11 | | 0.657 | [-0.60, 0.38] |  |
| Below mean motivation | 0.03 | 0.728 | [-0.15, 0.22] | 0.04 | 0.614 | [-0.12, 0.21] | 0.03 | | 0.736 | [-0.15, 0.21] |  |
| Constant | 2.88^***^ | 0.000 | [1.59, 4.17] | 2.86^***^ | 0.000 | [1.52, 4.20] | 2.91^***^ | | 0.000 | [1.63, 4.19] |  |
| R squared | 0.303 | | | 0.303 | | | 0.301 | | | |  |

Model 1 used assignment to treatment as the treatment variable (Intention-to-treat), model 2 used completion of at least four assignments as the treatment variable, and model 3 used assignment to the treatment group as an instrument for the treatment variable (Treatment-on-treated). GPA before treatment and educational track were included as controls. Models were estimated using robust standard errors. Models with standard errors clustered at the school level and with bootstrapped standard errors showed no differences. *p<0.05, **p<0.01, ***p<0.001. The number of observations was 294 in all models. GPA = Grade Point Average.

References used in supplementary material

van Boxtel, H. , Engelen, R., and de Wijs, A. (2011). Wetenschappelijke verantwoording van de Eindtoets 2010. (Scientific accountability exit test 2010), Arnhem: Cito.

CITO (2009), Terugblik en resultaten 2009 – Eindtoets basisonderwijs groep 8 (Review and results 2009 – exit test primary education grade 6), Arnhem: CITO.

Feron, E. (2018.) The role of cognitive tests and teachers in the transition from primary to secondary education. Chapter 5 Appendix C. PhD. Thesis Maastricht University School of Business and Economics: Maastricht.

Hurks, P. P. M. and Bakker, H. (2016). Assessing intelligence in children in youth living in the Netherlands. Int. J. School Educ. Psychol. 4:4, 266-275.

doi:10.1080/21683603.2016.1166754.

Verhaeghe, J. P., and J. Van Damme. 2007. “Leerwinst en toegevoegde waarde voor wiskunde, technisch lezen en spelling in eerste en tweede leerjaar.” SSL-rapport nr. OD1/05. Leuven: Steunpunt Studie- en Schoolloopbanen.

Zijsling, D., J. Keuning, H. Kuyper, Th. A. Van Batenburg, B. and Hemker. 2009. “Cohortonderzoek COOL5-18: Technisch rapport. Eerste meting COOL5-18 in het derde leerjaar voortgezet onderwijs.” Groningen: GION
